# Perioperative factors related to the prognosis of elderly patients with hepatocellular carcinoma

**DOI:** 10.1186/s40001-022-00896-5

**Published:** 2022-12-09

**Authors:** Lining Xu, Yingying Xu, Guiping Li, Bo Yang

**Affiliations:** 1 Department of General Surgery, The Second Medical Center & National Clinical Research Center for Geriatric Diseases, Chinese PLA General Hospital, Beijing, 100853 China; 2 Department of Internal Medicine, Henan Cancer Hospital, Zhengzhou, 450003 China; 3 Department of Radiology, Hubei Province Integrated Hospital of Chinese and Western Medicine, Wuhan, 430015 China; 4 Department of Radiology, Affiliated Union Hospital, Tongji Medical College, Huazhong University of Science and Technology, Wuhan, 430022 China

**Keywords:** Hepatocellular carcinoma, Elderly, Survival study, Hepatectomy, Perioperative

## Abstract

**Background:**

Hepatic resection is a potentially curative treatment for patients with hepatocellular carcinoma (HCC). The treatment of elderly patients with HCC has always been difficult. With the development of geriatrics and geriatric surgery, the number of elderly patients with HCC undergoing hepatectomy has gradually increased. To further improve the long-term prognosis of elderly patients with hepatocellular carcinoma undergoing surgery, it is necessary to study the related perioperative factors. Our aim was to study the impact of preoperative and intraoperative factors on the long-term survival of elderly patients with HCC who underwent hepatectomy.

**Methods:**

A total of 151 elderly patients with HCC who underwent hepatectomy were retrospectively studied. Univariate and multivariate Cox regression analyses were performed for preoperative- and intraoperative-related prognostic factors.

**Results:**

The 1-, 3-, 5- and 10-year overall survival rates of elderly patients with HCC who underwent resection were 79.5%, 60.8%, 46.6%, and 25.4%, respectively. Multivariate analyses identified four independent predictors of long-term prognosis: Child–Pugh grade (B/C versus A: HR[hazard ratio] = 2.318, *P* = 0.019), alpha-fetoprotein value (> 20 ng/ml versus ≤ 20 ng/ml: HR = 1.972, *P* = 0.005), resection style (anatomical versus no anatomical: HR = 1.976, *P* = 0.006), and intraoperative blood loss (> 400 ml versus ≤ 400 ml: HR = 2.008, *P* = 0.003).

**Conclusions:**

Poor survival of elderly patients with HCC who underwent hepatectomy was correlated with the preoperative and intraoperative factors of Child–Pugh grade, Alpha-fetoprotein value, resection style, and intraoperative blood loss.

## Background

In the past, the incidence of hepatocellular carcinoma (HCC) varied widely throughout the world, with high rates in sub-Saharan Africa, eastern and southeastern Asia, and Melanesia and a low incidence in Northern and Western Europe and America [1]. However, it is now rapidly becoming more prevalent in Western countries as a result of the spread of hepatitis C infection and the increased rate of liver cancer associated with alcohol use and nonalcoholic steatohepatitis [2]. HCC is, therefore, increasingly becoming a major contributor to the worldwide cancer burden. HCC is a histological type of primary liver cancer and is derived from hepatocytes [3]. In the 17th Nationwide Follow-up Survey of Primary Liver Cancer in Japan [4], 18,213 individuals were newly registered as patients with primary liver cancer at 645 medical institutions, and 94.2% of these patients had HCC. The incidence rates of this disease have increased in many countries in recent decades [5]. As the principal histologic type of liver cancer, HCC accounts for the great majority of liver cancer diagnoses and deaths.

Presently, hepatic resection is a potentially curative treatment for patients with malignant liver lesions, especially for HCC. The surgical outcome of HCC is affected by many factors [6]. How to optimize the perioperative factors of HCC to improve the surgical effect of HCC needs further research [7].

The world population is aging rapidly and the average life expectancy is higher than ever before. As a result, an increasing number of older individuals require surgical treatment. Older patients have low immunity, many perioperative comorbidities, degenerative changes in important organ functions, and reduced reserve and compensation capacities. Therefore, the prognosis of elderly patients with HCC undergoing hepatectomy is different from that of young people. Therefore, it is necessary to study the relationship between perioperative factors and prognosis in elderly patients with HCC, so as to provide new ideas for improving the surgical effect of elderly patients with HCC.

The present work retrospectively investigated 151 elderly patients with HCC who underwent hepatectomy and were followed up. This study aims to identify the impact of preoperative and intraoperative factors on the long-term prognosis of elderly patients with HCC who underwent hepatectomy.

## Materials and methods

### Patients

One hundred fifty-one elderly patients (≥60 years old) with HCC who underwent hepatectomy and subsequent follow-ups were enrolled in this investigation according to their medical documentations. Diagnoses came from pathological examination. The death of any patient within 30 days of hepatic resection or at any time after 30 days was considered a surgical death. These deaths and those that occurred during the hospitalization period for hepatectomy were excluded from our study.

Upon admission, all selected patients had records containing information from thorough disease histories, physical examinations, and so on. Preoperative data included patient demographics, diagnosis and laboratory blood analyses (bilirubin, alkaline phosphatase, etc.). Intraoperative data were collected from operation notes and the anaesthetists’ records and included operation times, estimated blood loss, resected regions, and other related items.

Blood transfusion during the operation was also recorded. Data regarding the resected specimen, such as the number of tumours and size of the largest tumour, were obtained from the pathology records.

The postoperative variables taken into consideration included complications and survival rates. Long-term outcomes (survival rates) were obtained through clinical follow-up and contact with the patient, in addition to family members if necessary.

This study was approved by the ethics committee of Chinese PLA General Hospital, and it did not contain any identification information about patients.

### Statistical analysis

All data are presented as the percentage of patients or as the mean with standard deviation. Survival was calculated. Plots were constructed according to the Kaplan–Meier method and compared with a log-rank test between groups. Cox regression was used to estimate the risk of death (hazard ratio, HR) for prognostic factors. A value of *P* < 0.05 was considered significant.

## Results

### Demographics

From January 1999 to December 2019, 151 elderly patients with HCC underwent hepatectomy and were followed up. These patients included 127 males and 24 females, and the average age was 63.85 ± 5.16 years (range 60–77). Tumours ranged from 1 to 19 cm in diameter. Tumours of 5–10 cm in size were found in 61 (40.4%) patients, while tumours > 10 cm were seen in 27 (17.9%) patients.

### Complications

The postoperative complications rate was 12.6%. The most common complications were related to liver cirrhosis, such as pleural effusion, ascites, and sterile perihepatic fluid collection. All complications were managed successfully by general treatment, such as concentrated human albumin transfusion, adjustment of water and electrolyte balance, or the use of diuretics.

### Patient survival

The 1-, 3-, 5- and 10-year overall survival rates of elderly patients with HCC who underwent resection were 79.5%, 60.8%, 46.6%, and 25.4%, respectively. Several preoperative and operative variables associated with survival rates in the univariate analysis are listed in Table 1, including sex (*P* = 0.040), alpha-fetoprotein (*P* = 0.000), total bilirubin (*P* = 0.001), Child–Pugh grade (*P* = 0.031), differentiation (*P* = 0.000), previous abdominal surgery (*P* = 0.013), resection style (*P* = 0.008), and intraoperative blood loss (*P* = 0.006). Multivariate analyses (Table 2) identified four independent predictors of long-term prognosis: Child–Pugh grade (B/C versus A: HR = 2.318, *P* = 0.019), alpha-fetoprotein value (> 20 ng/ml versus ≤ 20 ng/ml: HR = 1.972, *P* = 0.005), resection style (anatomical versus no anatomical: HR = 1.976, *P* = 0.006), and intraoperative blood loss (> 400 ml versus ≤ 400 ml: HR = 2.008, *P* = 0.003).

**Table 1:** Univariate analysis of factors associated with survival rate

Variable	Number	Survival rate (%)				*P* value^*^
		1-year	3-year	5-year	10-year	
Gender						
Male	127	76.4	57.3	41.3	19.0	*0.040*
Female	24	95.8	79.2	67.7	38.1	
Alpha-fetoprotein(ng/ml)^a^						
≤ 20	39	92.3	86.2	69.9	9.7	*0.000*
> 20	66	72.7	43.6	28.6		
Total bilirubin(μmol/L)^a^						
≤ 21	93	87.1	69.7	56.5	29.6	*0.001*
> 21	40	65.0	39.7	23.9		
Alkaline phosphatase(U/L)^a^						
≤ 130	99	79.8	60.4	48.3	21.6	*0.808*
> 130	21	50.6				
*HbsAg*^*a*^						
Negative	43	86.0	69.2	51.7		*0.056*
Positive	98	75.5	55.9	41.6	18.6	
Child–Pugh grade						
A	137	81.8	59.556.3	47.625.0	23.3	*0.031*
B/C	14	71.4				
Largest tumour size(cm)						
≤ 5	81	87.7	69.0	46.6	20.9	*0.052*
5 ~ 10	48	77.1	50.3	39.622.4		
> 10	22	63.6	40.9			
Number of lesion						
Single	111	81.1	63.8	49.3	23.9	*0.441*
Multiple	40	75.0	49.2	35.4	17.5	
Differentiation^a^						
Well differentiated	24	91.7	74.5	69.546.7	51.511.8	*0.000*
Moderately differentiated	64	82.8	66.8	17.5		
Poorly differentiated	33	54.5	30.0			
Previous abdominal surgery						
No	125	81.6	56.6	43.8	19.6	*0.013*
Yes	26	80.8	76.0	60.7		
≥ 3 segments resected						
No	111	81.1	63.8	49.3	23.9	*0.441*
Yes	40	75.0	52.5	39.4	17.5	
Resection style						
No anatomical	117	85.5	65.6	51.3	28.8	*0.008*
Anatomical	34	67.6	44.1	29.8	14.9	
Operating time(min)						
≤ 180	100	88.0	62.9	52.0	31.1	*0.064*
> 180	51	76.5	43.5	35.3	15.3	
Intraoperative blood loss(ml)						
≤ 400	114	89.5	63.8	51.0	32.8	*0.006*
> 400	37	67.6	51.4	30.8	5.5	
Intraoperative blood transfusion						
No	101	87.1	64.1	50.0	33.0	*0.065*
Yes	50	68.0	54.0	39.6	17.7	
Complication						
No	132	81.1	61.2	47.4	27.3	*0.739*
Yes	*19*	*68.4*	*39.4*	*19.7*		

^*^Log rank (Mantel–Cox) test

^a^Missing data

A value of P< 0.05 was considered significant.

**Table 2:** Multivariate analysis of factors associated with survival

Variable	HR	*P* value^*^
Child–Pugh grade (B/C versus A)	2.318	*0.019*
Alpha-fetoprotein value (> 20 ng/ml versus ≤ 20 ng/ml	1.972	*0.005*
Resection style (anatomical versus no anatomical)	1.976	*0.006*
Intraoperative blood loss (> 400 ml versus ≤ 400 ml)	2.008	*0.003*

^*^Cox regression

A value of P< 0.05 was considered significant.

## Discussion

### Importance and challenges of liver surgery in the elderly patients

The twenty-first century is an era of an aging population, and the proportion of older persons surgical patients is increasing every year. Extending human life is closely related to the progress of surgery. With careful perioperative care, the older persons may be successfully treated, and surgical complications and mortality will be reduced. We should emphasize active intervention of perioperative risk factors to improve the quality of perioperative diagnosis and treatment of older patients. Therefore, geriatric surgery has its unique considerations that are reflected in all aspects before, during, and after surgery. Perioperative factors are closely related to the prognosis of patients [8].

Some studies have shown that age is a relevant factor for survival in liver surgery. Perioperative factors have different effects on prognosis in different age stages. Therefore, this study aims to identify the impact of preoperative and intraoperative factors on the long-term prognosis of hepatocellular carcinoma elderly patients with HCC who underwent hepatectomy.

### Overall survival rates of elderly patients with HCC who underwent hepatectomy

The survival rates of hepatocellular carcinoma after liver resection are different among a variety of medical institutes. In Lee EC’s study [9], the rates of 1-, 3-, and 5-year overall survival were 91.9%, 78.9%, and 69.5%, respectively, and the rates of 1-, 3-, and 5-year recurrence-free survival were 71.7%, 51.7%, and 43.7%, respectively. One study compared the survival time after hepatectomy for hepatocellular carcinoma between the elderly and younger patients (79 elderly patients with age ≥ 70 years and 178 younger patients with age < 70 years.), the 1-, 3-, 5- and 7-year overall survival rates in the elderly and younger groups were 76%, 55%, 48%, and 42% and 79%, 57%, 51%, and 49%, respectively (*P* = 0.319). The 1-, 3-, 5-, and 7-year disease-free survival rates in the elderly and younger groups were 60%, 40%, 38%, and 27% and 54%, 36%, 32%, and 32%, respectively (*P* = 0.633) [10]. In our study, the 1-, 3-, 5- and 10-year overall survival rates of elderly patients with HCC who underwent resection were 79.5%, 60.8%, 46.6%, and 25.4%, respectively.

### Clinical characteristics associated with elderly HCC patients survival

Although advances in surgical techniques and perioperative management have reduced the incidence of complications and the mortality rate after hepatectomy, liver dysfunction after major hepatectomy remains an important problem. Liver failure after hepatectomy is the most common cause of death after liver surgery, and the decline in liver function in the older persons before surgery may be one of the reasons for this situation [11]. Therefore, a detailed preoperative assessment of liver function is important. The Child–Pugh grade is the most widely used evaluation method for liver function [12]. However, most of the current studies focus on the relationship between Child–pugh and complications, rarely on the relationship between Child–pugh and long-term prognosis. In this study, multivariate analysis indicated that Child–Pugh grade (B/C versus A: HR = 2.318, *P* = 0.019) was a prognostic indicator associated with a higher risk of death in the elderly patients. The resection style of the liver was an important factor associated with prognosis. It remains unclear whether hepatectomy for hepatocellular carcinoma should be performed as an anatomic resection or a nonanatomic resection. No randomized controlled trials are currently available on this topic. Jiao S [13] searched for articles about anatomic resection (AR) versus nonanatomic resection (NAR) for HCC published between January 1998 and December 2018 in the PubMed, Cochrane Library, EMBASE and Wanfang databases. Meta-analysis was performed on patient characteristics, tumour characteristics, operative characteristics, perioperative outcomes and long-term outcomes. A total of 38 studies involving 9122 patients were included. Of them, 5062 were in the AR group, and 4060 were in the NAR group. The AR group gained 1-, 3-, and 5-year overall survival and disease-free survival benefits versus the NAR group.

Our results were different from these findings. In this study, the 1-, 3-, 5- and 10-year survival rates in the no anatomical resection group were 85.5%, 65.6%, 51.3%, and 28.8%, respectively, and in the anatomical resection group they were 67.6%, 44.1%, 29.8%, and 14.9%, respectively. Multivariate analysis indicated that the survival rates in the no anatomical resection group were significantly higher than those in the anatomical resection group (*P* = 0.008). The advantages of no anatomical liver resection appear to be twofold: conserving liver function and reducing the dangers associated with more extensive liver resections. This is particularly advantageous in those patients who may be at higher risk for anatomical liver resections, such as those with cirrhosis and those who would be left with a small liver remnant. Some research has indicated that small-for-size livers promote tumour growth and metastasis [14, 15]. Liver regeneration after major surgery may activate occult micrometastases and facilitate tumour growth, leading to liver tumour recurrence [16].

Wong LL [17] reported that elevated alpha-fetoprotein levels, low levels of albumin and tumours > 5 cm in size were associated with increased 1-year mortality after hepatic resection for early-stage hepatocellular cancer in a retrospective study. Kondo K [18] reported that alkaline phosphatase (ALP, > 125 U/L), alpha-fetoprotein (AFP, within 20–400 or > 400 ng/mL), protein induced by vitamin K absence-II (PVIKA-II, within 40–400 or > 400 mAU/mL), tumour number, diameter, pseudocapsule, tumour growth pattern and intratumour haemorrhage were independent prognostic factors for hepatocellular carcinoma patients. In this study, multivariate analyses indicated that the alpha-fetoprotein value (> 20 ng/ml versus ≤ 20 ng/ml: HR = 1.972, *P* = 0.005) was an independent predictor of poor survival for HCC in the elderly patients.

There have been few reports of correlations between long-term survival and intraoperative factors, such as blood loss and blood transfusion. Several studies have suggested that preoperative blood loss or transfusions have a negative impact on postoperative outcomes. However, it has been debated whether this is due to a real cause-effect relationship or merely the result of a more complicated surgery. The effect of blood transfusions on tumour recurrence and long-term mortality is much less clear, and evidence varies depending on the type of malignancy [19]. In this study, multivariate analysis indicated that only intraoperative blood loss was a prognostic indicator associated with a higher risk of death (> 400 ml versus ≤ 400 ml: HR = 2.008, *P* = 0.003) in the elderly patients.

## Conclusions

In conclusion, the prognostic factors of HCC after hepatectomy are a controversial topic. Poor survival of HCC patients who underwent hepatectomy was correlated with the preoperative and intraoperative factors of Child–Pugh grade, Alpha-fetoprotein value, resection style, and intraoperative blood loss.

## Data Availability

All data are from the PLA General Hospital. The data will be made available on reasonable request. The datasets generated during and/or analysed during the current study are available from the corresponding author on reasonable request.

## References

[1] Srivatanakul P, Sriplung H, Deerasamee S (2004). Epidemiology of liver cancer: an overview. Asian Pac J Cancer Prev.

[2] Asrani SK, Devarbhavi H, Eaton J, Kamath PS (2019). Burden of liver diseases in the world. J Hepatol.

[3] Augustine MM, Fong Y (2014). Epidemiology and risk factors of biliary tract and primary liver tumors. Surg Oncol Clin N Am.

[4] Ikai I, Arii S, Okazaki M, Okita K, Omata M (2007). Report of the 17th nationwide follow-up survey of primary liver cancer in Japan. Hepatol Res.

[5] McGlynn KA, Petrick JL, El-Serag HB (2021). Epidemiology of hepatocellular carcinoma. Hepatology.

[6] Xu LN, Xu YY, Gao DW (2016). Impact of operative and peri-operative factors on the long-term prognosis of primary liver cancer patients undergoing hepatectomy. J Huazhong Univ Sci Technol (Med Sci).

[7] Walcott-Sapp S, Billingsley KG (2018). Preoperative optimization for major hepatic resection. Langenbecks Arch Surg.

[8] Trundle S, Gooneratne M, Rogerson A, Dhesi J (2019). Perioperative comprehensive geriatric assessment: what do we need to know?. Br J Hosp Med.

[9] Lee EC, Kim SH, Park H, Lee SD, Lee SA (2017). Survival analysis after liver resection for hepatocellular carcinoma: a consecutive cohort of 1002 patients. J Gastroenterol Hepatol.

[10] Hsu KF, Yu JC, Yang CW, Chen BC, Chen CJ, Chan DC, Fan HL, Chen TW, Shih YL, Hsieh TY, Hsieh CB (2018). Long-term outcomes in elderly patients with resectable large hepatocellular carcinoma undergoing hepatectomy. Surg Oncol.

[11] Lodewick TM, Alizai PH, van Dam RM (2017). Effect of age on liver function in patients undergoing partial hepatectomy. Dig Surg.

[12] Huang F, Gao J (2020). Modified Child-Pugh grade vs albumin-bilirubin grade for predicting prognosis of hepatocellular carcinoma patients after hepatectomy. World J Gastroenterol.

[13] Jiao S, Li G, Zhang D, Xu Y, Liu J (2020). Anatomic versus non-anatomic resection for hepatocellular carcinoma, do we have an answer? A meta-analysis. Int J Surg.

[14] Shoreem H, Gad EH, Soliman H, Hegazy O, Saleh S (2017). Small for size syndrome difficult dilemma: lessons from 10 years single centre experience in living donor liver transplantation. World J Hepatol.

[15] Kornberg A, Witt U, Kornberg J, Friess H, Thrum K (2015). Extended ischemia times promote risk of HCC recurrence in liver transplant patients. Dig Dis Sci.

[16] Li HM, Ye ZH (2017). Microenvironment of liver regeneration in liver cancer. Chin J Integr Med.

[17] Wong LL, Hernandez BY, Shvetsov YB, Kawano Y, Tang ZY (2016). Liver resection for early hepatocellular cancer: comparison of centers in 3 different countries. World J Hepatol.

[18] Zhou Q, Zhou C, Yin Y, Chen W, Liu C (2021). Development and validation of a nomogram combining hematological and imaging features for preoperative prediction of microvascular invasion in hepatocellular carcinoma patients. Ann Transl Med.

[19] Bennett S, Baker LK, Martel G, Shorr R, Pawlik TM (2017). The impact of perioperative red blood cell transfusions in patients undergoing liver resection: a systematic review. HPB.

